# A simple and rapid RP-HPLC method for the assessment of cobalamin (vitamin B12) in tilapia and snoek fishes

**DOI:** 10.55730/1300-0527.3309

**Published:** 2021-10-13

**Authors:** Labaran IBRAHIM, Aminu USMAN

**Affiliations:** 1 Department of Biochemistry, Faculty of Science, Federal University Duste, Jigawa State, Nigeria; 2 Department of Biochemistry, Faculty of Natural and Applied Sciences, Umaru Musa Yar’adua University, Katsina State, Nigeria

**Keywords:** Isocratic, precise, stationary phase, chromatography, retention time, limit of detection

## Abstract

This study developed a rapid reversed-phase high-performance liquid chromatography (RP-HPLC) method equipped with a UV-Vis detector. The aquaponics system›s tilapia and marine snoek fishes were extracted using an autoclaving process and enzymatic treatment. The technique enabled the separation and quantification of cobalamin present in these fishes extracts. Phenomenex Luna^®^ 5 μm C18 (2) 100 A LC-column (150 × 4.6 mm) was used as a stationary phase, while the mobile phase consisted of methanol and phosphoric acid in a ratio of 35:65 (v/v), respectively. The developed method was revealed to be rapid (a retention time of less than 5.0 min), linear (R^2^ = 0.9988), sensitive (limits of detection and quantification showed to be 0.0004 and 0.0011 μg/mL, respectively), precise (percentage relative standard deviation of 0.14% to 0.76%), and accurate (percentage mean recovery of 87.44 ± 0.33% to 97.08 ± 0.74%). The quantified concentrations of this vitamin in extracts of tilapia and snoek fishes were found to be 08.95 ± 0.35 and 14.97 ± 0.04 μg/mL, respectively. Therefore, we suggested that the developed RP-HPLC technique could be applicable for quality control evaluation in the food and pharmaceutical industries. Besides, the method could be vital for diagnostic analysis in clinical laboratories.

## 1. Introduction

Vitamins are organic chemical compounds of large categories required in minor concentrations for normal immune and catalytic functions, body growth, repair, and development [1,2]. Vitamin deficiency-related physiological disorders or abnormalities are associated with their inadequate amounts in diets [3]. Night blindness and mental retardation are examples of diseases associated with vitamins deficiency, and this depends on which specific vitamin is absent or deficient [4]. For instance, Nicotinamide (vitamin B_3_) is essential for carbohydrate metabolism and DNA repair by transfer reactions of nonredox adenosine diphosphate-ribose [4]. Pyridoxine (vitamin B_6_) plays a vital role in riboflavin activity as a coenzyme for different respiratory enzymes and transamination of amino acids [4].

Cobalamin (vitamin B_12_) is a water-soluble vitamin with cobalt as a central atom. Cyanocobalamin is another popular name for cobalamin. The name applies to any of the cobalamins with identical biological functions [5]. It existed in 4 different forms: methylcobalamin, hydroxocobalamin, cyanocobalamin, and 5′-deoxyadenosylcobalamin [6,7].

Cyanocobalamin is the nonnatural form of this vitamin used for food product fortification due to its high stability [6]. Production of cobalamin is mainly by microorganisms [6]. Nevertheless, it can be naturally present in animal-derived food substances like liver, meat, egg, and synthetic form as in dietary supplements [6,8]. However, cobalamin is not mostly readily available in materials of plant origin [6]. From the report of [9], this vitamin plays a vital role in homocysteine balance, a risk factor for arteriosclerosis. In addition, cobalamin serves as a coenzyme for many essential enzymes in one-carbon (methyl) metabolism, involved in human growth and development [10]. Moreover, it is significant for hemoglobin synthesis and for normal functioning of the brain and nervous system [5]. The daily recommendation of cobalamin is 1μg. Due to its low absorption rate in the small intestine, 3 μg amount may be required [10]. In normal populations, the deficiency of this vitamin is rare. Nevertheless, eating disorders, elderly individuals, vegetarians or vegans, infants, and pregnant women can be susceptible to its deficiency because of their physiological needs and prescribed dietary requirements [11]. As recommended by the American Dietary Guideline (ADG), a cobalamin dietary supplement can be adequate for individuals at risk of its deficiency as an alternate food nutrient source [12].

This vitamin occurs in an accessible free form or bound to other molecules such as protein. If bound, it can be released by heating at 98 °C for 30 min with potassium cyanide as reported by [13], or through autoclaving at 121 °C using buffer solution and excess potassium cyanide [14]. Samples treatment with α-amylase for 3 h at 37 °C and incubation for 35 min at 100 °C is another possible process for its release [15].

From the report of [16], a microbiological assay using *Lactobacillus leishmania* could be another applicable method of this vitamin analysis. The microbial assay method usually has a low specificity in some food matrices and is time-consuming [15,17]. Also, diverse reverse-phase high-performance liquid chromatography (RP-HPLC) methods for the evaluation of this water-soluble vitamin were reported in milk [18], meat product [6], okra [19], dietary supplement, and ingredients [16,20,21] as well as blood serum [22].

Nevertheless, there is a scarcity of data in published literature for cobalamin assessment in fishes using the isocratic RP-HPLC method. Therefore, in this study, we intended to determine the level of this vitamin from the extracts of aquaponics tilapia and marine snoek fishes using the RP-HPLC method with a UV-vis detector.

## 2. Materials and methods

The purchase of all samples for our study work was on 15.01.2021. The acquired fresh aquaponics tilapia and marine snoek fishes were from Sabon Gari market situated in Fagge Local Government, Kano State, Nigeria. The transportation of each sample was in separate clean polyethylene bag to the laboratory for experimental analysis. Each sample was separately cleaned well with distilled water, dried using a blotting paper, and stored at −20 °C before analysis.

**Figure 1:**
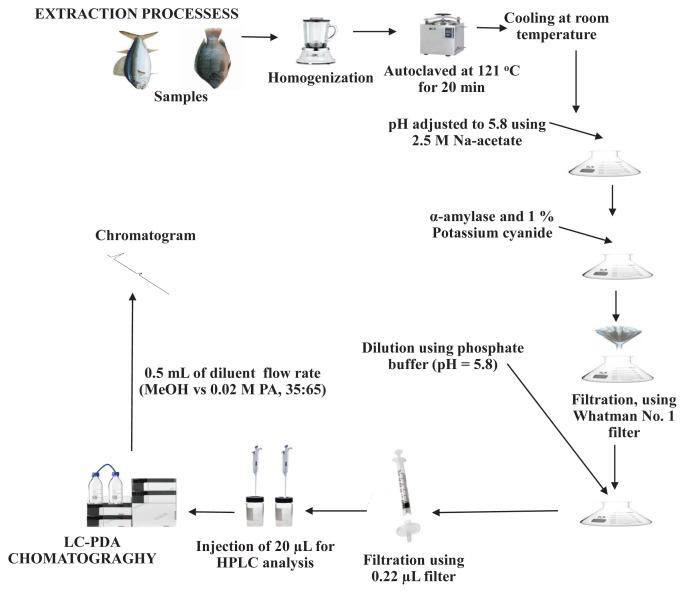
Schematic diagram of samples extraction processes and chromatography.

Various extraction processes of cobalamin were previously reported in [15,23]. In this study, the extraction process for the release of the cobalamin from each fish sample was carried out using acidic autoclaving and enzymatic treatment for its proper separation from other components and to obtain a maximum yield, respectively. Each fish sample was chopped out separately into pieces and homogenized using a Waring blender. To approximately 10 g of each homogenized fish sample, 100 mL of sulfuric acid solution (0.1 Eq/L) was added, autoclaved at 121 °C for 20 min. The cooling of each autoclaved sample extract was at room temperature, and the pH was adjusted to 5.8 using 2.5 M sodium acetate solution. Besides, α-amylase (70 mg) and 1% potassium cyanide (2 mL) were added, vortexed properly, and kept overnight at room temperature. Each extract was then filtered using a filter paper (Whatman No. 1). The dilution of each filtrate was by using a phosphate buffer, pH = 5.8 (diluting solution) in a ratio of 1:2 (v/v), respectively. Finally, the filtration of each diluted fish extract was through a micropore syringe filter (0.22 μm) into amber HPLC vials for chromatography. All reaction vessels were covered with aluminum foil during the extraction processes to prevent photo-induced degradation of analytes. Figure 1 illustrates the extraction processes of cobalamin from the study samples.

The preparation of a standard (cyanocobalamin) stock solution was as previously described by [24], with a modification in the preparation of working standard concentrations. The preparation of the stock solution was by dissolving 10 mg of cyanocobalamin in 25 mL of a diluting solution (phosphate buffer, pH = 5.8). A standard working solution of 200 μg/mL was generated using the standard stock solution to generate 0.1. 0.25, 0.5, 1, 2.5, 15, 20, 50, and 120 μg/mL concentrations for further dilution using diluting solution.

The purchased external standard (cyanocobalamin) and α-amylase were from Sigma-Aldrich, St. Louis, USA. The sourced HPLC grade methanol was from Merck KGaA, Darmstadt, Germany, CAS-No. 67-56-1. The acquired orthophosphoric acid and sulfuric acid were from Merck Chemicals (PTY) LTD, Germany, and Minema, South Africa, Batch No. 42980, respectively. The obtained Milli-Q water was from the EMD-Millipore machine (Switzerland). Potassium hydroxide, mono, and dibasic potassium hydrogen phosphate, sodium acetate, and potassium cyanide were provided by Merck Laboratory Supplies (PTY), Midrand, South Africa. The purchased filter disc Grade 292 (125 mm) was from Whatman, Maidstone, England, while Minisart Syringe Filter (0.22 μm) was from Bornstein, Germany. All solvents, materials, and reagents used for this research work are of analytical grade.

The HPLC-Shimadzu-UFLC Prominence system with the LC-20AD connecter, LC-2AB pump (20 MPa), SIL-2A autosampler, and SPDA-M20A diode array detector was from Shimadzu Corporation, Kyoto, Japan. The PDA wavelength of the detector was between 190 to 800 nm. The “LC Lab Solution” software performed the system’s control, data acquisition, and analysis.

The conducted chromatographic separation was by using the developed method on Luna^®^, 5μm C18 (2) 100A column (150 × 4.6 mm (Phenomenex, USA). The mobile phase delivery was isocratic and consisted of methanol (MeOH) and 0.02 M phosphoric acid (PA), pH = 5.8 in a ratio of 35:65, v/v (A/B), respectively. The flow rate was 0.5 mL/min.

The maintained column temperature was 30 °C, and each analyte injection volume was 20 μL for 10 min run. The recorded Ultraviolet-visible (UV-vis) absorbances were at 270 nm wavelength [25].

## 3. Results and discussion

Cobalamin occurs in diverse natural conformation. Therefore, transformation into a single conformation like cyanocobalamin is required. The extraction processes yield this conversion and hence, its identification using a UV-vis detection wavelength of 270 nm. However, the often-used UV detection wavelength by the AOAC official method was 360 nm [26]. During the optimization of our developed analytical method, both 270 and 360 nm wavelengths were evaluated. A wavelength of 270 nm produced a better peaks resolution for the detection of cobalamin. Also, the wavelength yielded a reasonable level of this vitamin. Generally, extraction of B-vitamins can be possible by incubating a sample at 121 °C for 20 min in the presence of excess potassium cyanide [6]. From the report of [18], the acid digestion and addition of α-amylase enzyme are significant enough for sample extraction of vitamin B-complexes. For our samples’ extractions, both approaches were exercised for maximum yield. Therefore, RP-HPLC using a UV-vis detector is one of the accepted assay methods for separating, identifying, and quantifying vitamins and other organic compounds in pharmaceutical supplements and food formulas [27]. In addition, from the report of [28], the RP-HPLC analysis method coupled to a UV-vis detector was the most reliable, fast, and simple technique for studying water-soluble vitamins in food materials.

The method validation of our developed RP-HPLC complied with the international conference of harmonization (ICH) [29]. The linearity, sensitivity, detection limit (LOD) and quantification limit (LOQ), precision, and accuracy were the characteristics parameters evaluated during our method validation. The analysis of all working solutions was within 24 h of their preparation.

**Figure 2:**
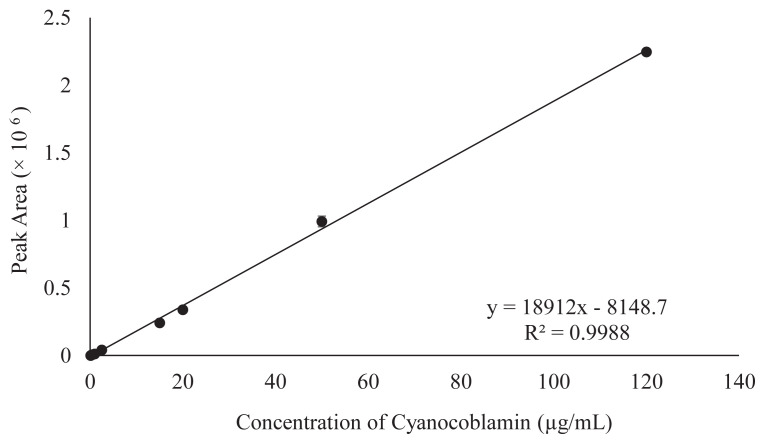
Standard linear regression curve of standard having a 9-points range of concentrations (0.5 to 120 μg/mL). The y-axis represents the peak area of chromatograms in mAU. However, the x-axis represents the standard (Cyanocobalamin) concentration in μg/mL.

The linear regression graph analysis was performed in duplicate under a 9-point calibration graph to determine the linearity of the proposed method. The observed response was linear within the above concentration ranges of the standard working solution. The revealed coefficient (R^2^) of the linear regression graph and equation were 0.9988 and y = 18912x – 8148.7 (x = concentration, y = peak area), respectively (Figure 2).

The LOD and LOQ for the developed method were evaluated based on a signal by comparing 3.3-folds and 10-folds differences of the baseline noise and signal of analytes, respectively. The LOD means a concentration of compound that produces a signal-to-noise ratio of above 3.0. However, the LOQ means a compound concentration equal to 10 times the value of the signal-to-noise ratio. The LOD and LOQ were determined using these formulas; 3.3 × (S)/m) and 10 × (S)/m) respectively, as cited by the ICH guidelines [29]. Here S is standard deviation of y-intercept, and m is slope of the linear regression curve. The determined LOD and LOQ were 0.0004 and 0.0011 μg/mL, respectively (Table 1). Hence, the LOD and LOQ concentration values determined indicated that the method developed was of high sensitivity.

The assessed method precision (intraday and interday) was by using 2-different concentrations (5 and 10 μg/mL) of the standard working solution. However, within points in the standard curve, they were not identified. The evaluation of each concentration was in duplicate, and each result presented as a relative standard deviation (RSD) in percentage, which denotes average recovered standard deviation/average recovered concentration × 100. The evaluation of intraday and interday precision were within 24 h and 48 h, respectively. The recorded interday and intraday values of the precision were in the range of 0.14% to 0.76% (Table 2). Similarly, the evaluation for intraday and interaccuracy was in duplicate using the same standard solution concentrations used to determine intraday and interday precision. The presentation of each data for intraday and interaccuracy was as a percentage mean recovery and recorded between 87.44 ± 0.33% to 97.08 ± 0.74%, respectively, as depicted in Table 2. Therefore, the realized low values of the percentage RSD, ≤2.5 as recommended by AOAC, and satisfactorily percentage mean recoveries revealed a high precision and accuracy of the developed isocratic RP-HPLC method for assessing this vitamin.

**Figure 3:**
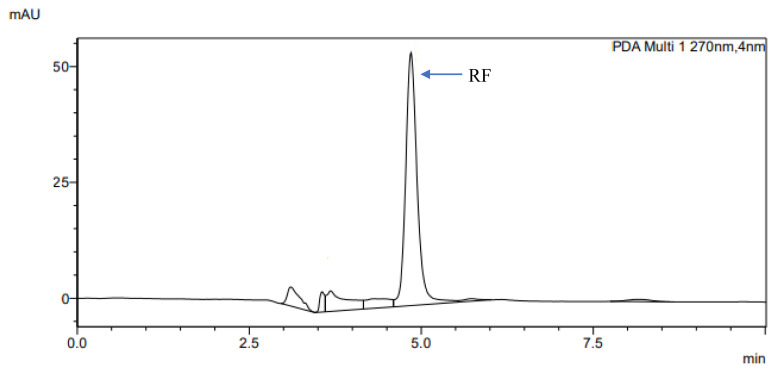
The representative chromatogram for the RS (30 μg/mL).

**Figure 4:**
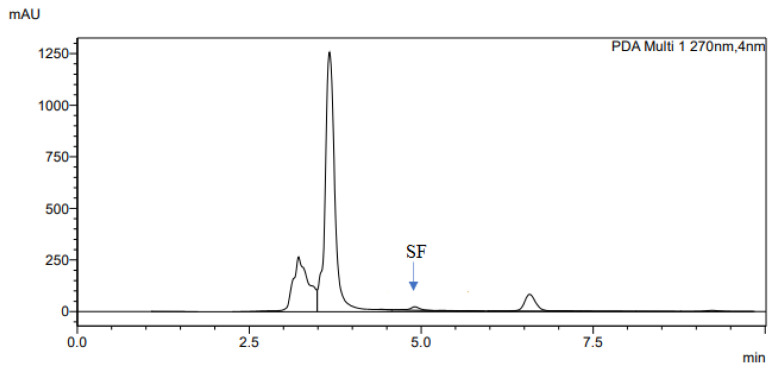
The representative chromatogram for the SF.

**Figure 5:**
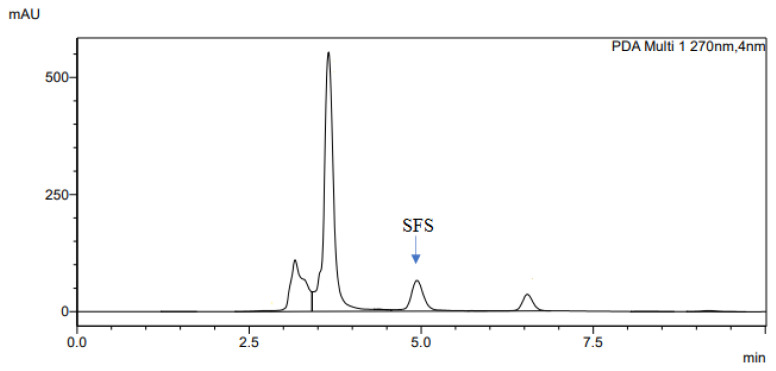
The representative chromatogram for the SFS.

**Figure 6:**
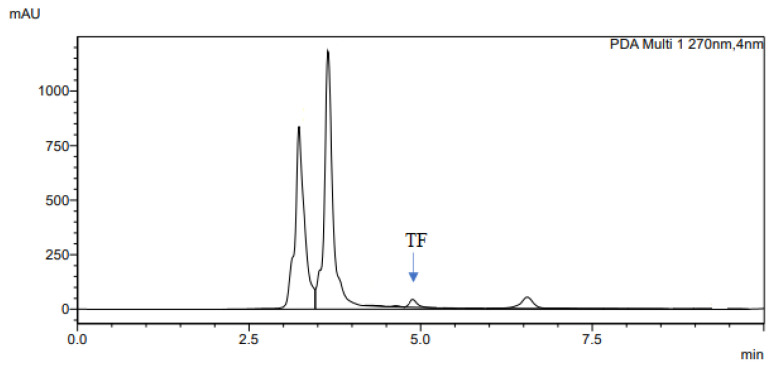
The representative chromatogram for the TF.

**Figure 7:**
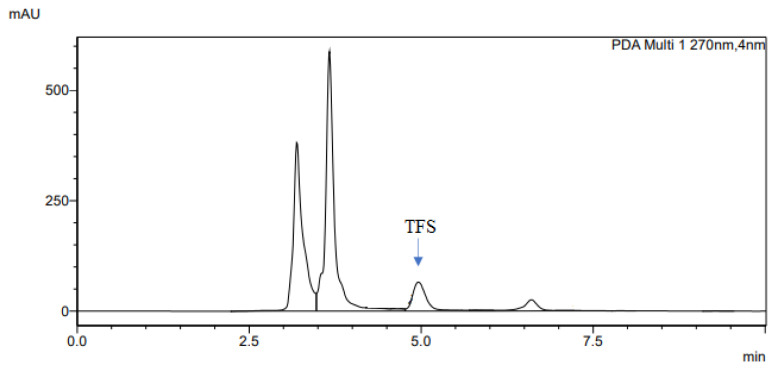
The representative chromatogram for the TFS.

**Figure 8:**
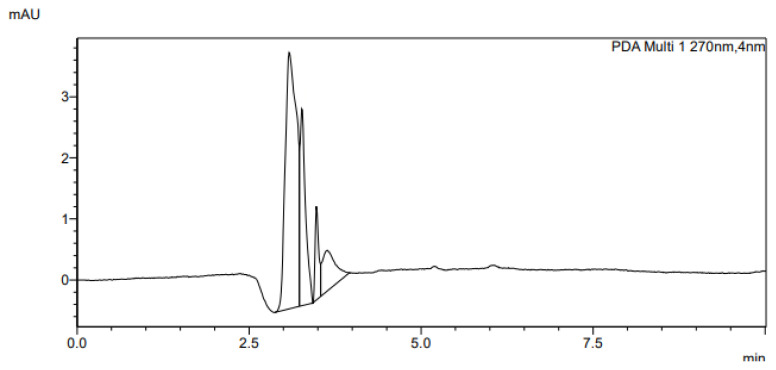
The representative chromatogram for the DS. The above UV-HPLC chromatograms were detected using UV-detector with an isocratic gradient of 35% methanol and 65% weak phosphoric acid (0.02 M). The flow rate was 0.5 mL/min, the injection volume was 20 μL, and the detection wavelength was 270 nm wavelength.

The determination of accuracy-spike recovery was by assaying each extract of a snoek and tilapia fishes fortified with a known concentration of standard cyanocobalamin called a reference standard in a ratio of 1:1 (v/v). The representative chromatograms of the reference standard (RS), snoek fish (SF) (unspiked extract), snoek fish spiked (SFS) extract, tilapia fish (TF) (unspiked extract), tilapia fish spiked (TFS) extract, and diluting solution (DS) are presented in Figures 3–8, respectively. The determination of the percentage accuracy-spike of each sample extract was by using the relation below. The expression of each result was as a percentage mean recovery (Table 3). Therefore, % MR = ((A – B)/C)) × 100, where A, B, and C and % MR denotes peak area of the spiked sample extract, the peak area of the unspike sample extract, the peak area of the reference standard, and percentage mean recovery.

Each extract quantification of cobalamin (Table 3) was expressed in μg/mL and calculated using a linear regression equation, y = 18912x – 8148.7 (where, x = concentration, y = peak area). The amount of cobalamin obtained from this study was compatible with meat products’ reported work [6]. It was higher than the amount revealed using okra extracts from 4 different geographical locations as reported in [19]. The recommended nutrient intake of cobalamin by US-NAS (United States National Academy of Science) ranges between 0.4 and 2.8 μg/day for all age groups, including pregnant and lactation mothers. However, older individuals may require a higher amount per day [30]. From levels of cobalamin obtained from our study, the sample extracts, therefore, denoted good dietary sources of this vitamin.

The retention times of various chromatograms presented in Figures 3–8 and the consistency of our developed RP-HPLC method with previously cited ones were given in Tables 4 and 5, respectively. Therefore, our method was revealed to be even more precise (% RSD), sensitive (LOD and LOQ), and rapid (retention time) when compared with previous findings.

## 4. Conclusion

The isocratic liquid chromatography method we developed for this research study was simple, rapid, linear, sensitive, precise, and accurate. Moreover, from the results obtained, our extraction method indicated that the extraction and separation of this vitamin were satisfactory.

Therefore, the developed method can be vibrant to the food and pharmaceutical industries for routine product quality control and nutritional labeling. Also, the method could be of importance to clinical laboratories for diagnostic analysis. Moreover, data obtained could be applicable as food-based dietary guidelines, nutrition education, and a food composition database.

## Figures and Tables

**Table 1:** Characteristics of the isocratic RP-HPLC method. Values presented were as mean ± SD in duplicate.

Characteristic parameters	Cobalamin (Vitamin B_12_)
Calibration range of standard, μg/mL	0.1 to 120
Correlation coefficient, R^2^	0.9988
Linear regression equation	y = 18912x – 8148.7
LOD a, μg/mL	0.0004
LOQ b, μg/mL	0.0011
Wavelength of detection, nm	270

a LOD, Limit of detection and

b LOQ, Limit of quantification

**Table 2:** The precision and accuracy of the developed isocratic RP-HPLC method; values presented were as means ± SD in duplicate.

Content, μg/mL	Intraday		Interday	
	Precision (RSD a ), %	Accuracy (Recovery), %	Precision (RSD a ), %	Accuracy (Recovery), %
5	0.76	97.08 ± 0.74	0.36	96.86 ± 0.35
10	0.14	90.20 ± 0.12	0.37	87.44 ± 0.33

a RDS, relative standard deviation

**Table 3:** Accuracy-spiked and measured samples extract concentrations of cobalamin. Values presented were as means ± SD in duplicate.

Name of samples	Accuracy-spike level, %	Concentration of Cobalamin, μg/mL
Aquaponics tilapia fish	102.59 ± 0.09	14.97 ± 0.04
Marine snoek fish	110.84 ± 1.18	08.95 ± 0.35

**Table 4:** Analytes chromatograms’ retention times. Values presented were as mean ± SD in duplicate.

Analytes	Retention time, min
RS	4.98 ± 0.19
SF	4.92 ± 0.01
SFS	4.95 ± 0.01
TF	4.89 ± 0.03
TFS	4.95 ± 0.02

RS; reference standard, SF; snoek fish extract, SFS; snoek fish spiked, TF; tilapia fish extract, TFS; tilapia fish spiked.

**Table 5:** Various RP-HPLC methods consistency-comparison for the determination of B-vitamins.

S/N	Methods properties	Values reported
1.	Regression (R^2^)	0.9988
	LOD, μg/mL	0.0004
	LOQ, μg/mL	0.0011
	Precision (RSD), %	0.76–0.36
	Percentage recovery, %	87.44–90.20
	Retention time, min	4.87–4.98
	Detection wavelength, nm	270
	Detected level, μg/mL	8.95–14.97
	Sample extracts	Fishes (Tilapia and Snoek)
	Reference	[*]
2.	Regression (R^2^)	0.9950
	LOD, μg/mL	0.2000
	LOQ, μg/mL	0.3800
	Precision (RSD), %	nr
	Percentage recovery, %	nr
	Retention time, min	16.00
	Detection wavelength, nm	210
	Detected level, μg/mL	nr (lower than LOD)
	Sample extracts	Polyvitaminated premixes
	Reference	[15]
3.	Regression (R^2^)	0.9990
	LOD, μg/mL	nr
	LOQ, μg/mL	0.007
	Precision (RSD), %	1.50–7.26
	Percentage recovery, %	79.61–108.80
	Retention time, min	7.17
	Wavelength, nm	361
	Detected level, μg/mL	3.85–8.78
	Sample extract	Meat products
	Reference	[6]
4.	Regression (R^2^)	0.9910
	LOD, μg/mL	0.0625
	LOQ, μg/mL	0.1250
	Precision (RSD), %	0.40–4.10
	Percentage recovery, %	90.40–108.50
	Retention time, min	6.53
	Wavelength, nm	350
	Detected level, μg/mL	45.86
	Sample extracts	Multivitamin tablets
	Reference	[20]
5.	Regression (R^2^)	0.9990
	LOD, μg/mL	0.1600
	LOQ, μg/mL	0.5200
	Precision (RSD), %	1.41–4.64
	Percentage recovery, %	96.00–10.10
	Retention time, min	5.70
	Detecttion wavelength	228
	Detected level, μg/mL	1.80–2.69
	Sample extract	Dietary supplements and ingredients
	Reference	[21]

[*] = Our developed RP-HPLC method.
